# Persistent NRG1 Type III Overexpression in Spinal Motor Neurons Has No Therapeutic Effect on ALS-Related Pathology in SOD1^G93A^ Mice

**DOI:** 10.1007/s13311-023-01424-x

**Published:** 2023-09-21

**Authors:** Sara Hernández, Sara Salvany, Anna Casanovas, Lídia Piedrafita, M. Clara Soto-Bernardini, Olga Tarabal, Alba Blasco, Sílvia Gras, Alaó Gatius, Markus H. Schwab, Jordi Calderó, Josep E. Esquerda

**Affiliations:** 1 Unitat de Neurobiologia Cel·lular, Departament de Medicina Experimental, Facultat de Medicina, Universitat de Lleida and Institut de Recerca Biomèdica de Lleida (IRBLleida), Lleida, Catalonia Spain; 2 Department of Neurology, University Hospital Leipzig, Leipzig, Germany; 3 Center for Research in Biotechnology (CIB), Costa Rica Institute of Technology (TEC), Cartago, Costa Rica; 4 Paul Flechsig Institute of Neuropathology, University Hospital Leipzig, Leipzig, Germany

**Keywords:** Amyotrophic lateral sclerosis, ALS, Neuregulin, NRG1, Motor neuron, SOD1, Therapy

## Abstract

Amyotrophic lateral sclerosis (ALS) is a devastating neurodegenerative disease affecting upper and lower motor neurons (MNs). Neuregulin-1 (NRG1) is a pleiotropic growth factor that has been shown to be potentially valuable for ALS when supplemented by means of viral-mediated gene therapy. However, these results are inconsistent with other reports. An alternative approach for investigating the therapeutic impact of NRG1 on ALS is the use of transgenic mouse lines with genetically defined NRG1 overexpression. Here, we took advantage of a mouse line with NRG1 type III overexpression in spinal cord α motor neurons (MN) to determine the impact of steadily enhanced NRG1 signalling on mutant superoxide dismutase 1 (SOD1)-induced disease. The phenotype of SOD1^G93A^-NRG1 double transgenic mice was analysed in detail, including neuropathology and extensive behavioural testing. At least 3 animals per condition and sex were histopathologically assessed, and a minimum of 10 mice per condition and sex were clinically evaluated. The accumulation of misfolded SOD1 (mfSOD1), MN degeneration, and a glia-mediated neuroinflammatory response are pathological hallmarks of ALS progression in SOD1^G93A^ mice. None of these aspects was significantly improved when examined in double transgenic NRG1-SOD1^G93A^ mice. In addition, behavioural testing revealed that NRG1 type III overexpression did not affect the survival of SOD1^G93A^ mice but accelerated disease onset and worsened the motor phenotype.

**Supplementary Information:**

The online version contains supplementary material available at 10.1007/s13311-023-01424-x.

## Introduction

ALS is a disease affecting upper and lower MNs in the cerebral cortex, brain stem, and spinal cord, leading to progressive muscle paralysis and death. There is no effective treatment for halting or significantly delaying the disease [1]. Mouse models of ALS expressing mutant superoxide dismutase 1 (SOD1) have been extensively used for preclinical drug search. However, the translational value of most of these findings for the development of human therapies has been challenged owing to their poor results in clinical trials [2]. The enormous complexity of mechanisms underlying ALS pathogenesis accounts for this limited translational success. Nevertheless, ALS rodent models represent an important tool to examine ALS pathophysiology and to assist in the development of future therapeutic strategies for this disease [3–6].

Several neurotrophic factors have been tested as therapeutic agents in preclinical studies. Among them, vascular endothelial growth factor (VEGF), glial-derived neurotrophic factor (GDNF), ciliary neurotrophic factor (CNTF), and insulin-like growth factor 1 (IGF-1) exhibited some positive impact in mouse models, but so far, this has not been successfully verified in clinical trials [7]. Another growth factor that has been tested in SOD1 ALS mice is neuregulin-1 (NRG1), an epidermal growth factor (EGF)-like domain containing ligand for transmembrane tyrosine kinase receptors of the ErbB family. NRG1 serves pleiotropic regulatory functions in the nervous system, including myelination and synaptogenesis [8–11]. Variants of the human *ERBB4* gene, coding for the main NRG1 receptor in the central nervous system (CNS), have been identified as causative of a new form of familial ALS [12]. In spinal α-MNs, NRG1 is concentrated at postsynaptic sites facing cholinergic presynaptic terminals (C-boutons), in which ErbB receptor expression has been identified; this suggests a retrograde NRG1-ErbB signalling axis with functional relevance in these synapses [13, 14]. C-boutons are involved in the regulation of α-MN excitability during locomotor behaviour [15–17] and modulate their vulnerability in ALS [18]. In fact, several reports have described alterations of C-boutons during ALS [18–21] (see also [22]), including loss of its associated NRG1 [13, 23]. Thus, C-boutons have been suggested as potential pharmacological targets for ALS therapy [24, 25]. Accordingly, viral-mediated delivery of NRG1 type III (the most abundant NRG1 isoform in α-MNs) into the spinal cord restored the number of C-boutons and prolonged the lifespan of SOD1^G93^^A^ mice [23, 26], but the cellular distribution of virally expressed NRG1 was not analysed in detail. Moreover, contradictory data showing a lack of therapeutic actions of NRG1 after intraventricular administration and even neuroprotective effects of blocking endogenous NRG1 signalling [27] further add to the ill-defined value of NRG1-based treatment strategies in SOD1^G93A^ mice. Consequently, the mechanisms involved in the observed inconsistent activities of NRG1 in the disease course remain inconclusive.

A genetically well-defined alternative approach for investigating the impact of augmenting NRG1 signalling on ALS-relevant pathology in the SOD1^G93^^A^ model is the use of transgenic mouse lines with thymocyte differentiation antigen 1.2 promoter-driven neuronal overexpression of distinct NRG1 isoforms. These mouse models have been used to study axonal control mechanisms during myelination of spinal MNs [28, 29]. We have previously reported that transgenic overexpression of distinct NRG1 isoforms has a robust differential impact on pre- and post-synaptic elements of C-boutons in MNs [30].

Here, we took advantage of one of these mouse lines to determine how steady overexpression of NRG1 type III, the predominant NRG1 isoform in α-MNs, interacts with SOD1-induced disease mechanisms in double transgenic NRG1-SOD1^G93^^A^ mice. Our results indicate that NRG1 type III overexpression greatly influences the organization of endoplasmic reticulum (ER)-related organelles, such as the subsurface cistern (SSC), in MNs, and promotes peripheral nerve myelination. However, this approach has no beneficial impact on the clinical development of ALS. A similar strategy was recently applied in a mouse model of spinal muscular atrophy, resulting in an amelioration of some phenotypic parameters but without a subsequent improvement of lifespan [31].

## Material and Methods

### Animals, Genotyping, and Breeding

Transgenic mice used in this study were as follows: (1) ALS mouse model: B6SJL-Tg (SOD1^G93^^A^) 1Gur/J (SOD1^G93A^) mice (referred to as SOD1^G93A^) obtained from Jackson Laboratory (Bar Harbor, ME, USA) with overexpression of human SOD1-G93A (named “fast” [SOD1^G93A^-f]), resulting in a mean lifespan around 160 days (LIT) [32, 33]; and (2) NRG1 transgenic mice: C57Bl6-Tg (Thy1-Nrg1*III)1Kan+/− that overexpress an N-terminally HA epitope-tagged NRG1 type III isoform (hereafter referred to as NRG1) under control of the Thy1.2 promoter, which is active in postnatal MNs [29]. Transgenic lines were maintained as hemizygotes by crossing transgenic males with non-transgenic females on a B6 background. For the generation of double transgenic NRG1-SOD1^G93A^ mice, NRG1 type III females and SOD1^G93A^ males were crossbred. Animals were housed in the Animal Facility of *Universitat de Lleida* and kept in a strictly controlled environment (12-h light/dark cycle and 20 ± 2 °C of room temperature). Chow and water were provided ad libitum.

Genomic DNA was extracted from a tail biopsy using the Phire Kit (ThermoFisher Scientific, Waltham, MA). The genotype of the offspring was identified using PCR with the following primers: 5′-GGCTTTCTCTGAGTGGCAAAGGACC-3′ for the forward HANI-Nrg1 transgene and 5′-GTCCACAAATACCCACTTTAGGCCAGC-3′ for reverse HANI-Nrg1 transgene. For SOD1 genotyping, the following primers were used: IMRO 113, 5′-CATCAGCCCTAATCCATCT-3′ and IMRO 114, 5′-CGCGACTAACAATCAAAGTGA-3′.

All animal experimentation procedures were performed according to the European Committee Council Directive, the Animal Care and Use and Biosecurity Committees of the *Universitat de Lleida*, and the norms established by the *Generalitat de Catalunya* (*Diari Oficial de la Generalitat de Catalunya (DOGC) 2073, 1995*).

### Tissue Sample Preparation, Immunohistochemistry, and Imaging

Animals were deeply anesthetized with inhaled isoflurane and transcardially perfused with a physiological saline solution followed by 4% paraformaldehyde (PFA) in 0.1 M phosphate buffer (PB) at pH 7.4. The lumbar spinal cord and L4 ventral roots were dissected. Spinal cord samples were then postfixed for 24 h at 4 °C in the same fixative solution and then cryoprotected at 4 °C with 30% sucrose in 0.1 M PB containing 0.02% sodium azide. Transverse cryostat sections (16 µm thick) were collected on gelatine-coated glass slides.

Cryostat sections were permeabilized with phosphate-buffered saline (PBS) containing 0.1% Triton X-100 for 30 min, blocked with either 10% normal goat serum or normal horse serum in PBS for 1 h at room temperature (RT), and then incubated overnight at 4 °C with an appropriate primary antibody mixture. The primary antibodies used are as follows: ionized calcium-binding adapter molecule 1 (Iba1, 1:500, goat polyclonal, Abcam (ab5076)), CD68 (rat anti-mouse CD68,1:500 Bio-Rad MCA1957T), SOD1 (C4F6) Anti-misfolded human SOD1 (1:100, mouse monoclonal, MediMabs 2B Scientific (MM-00070-2-P)), glial fibrillary acidic protein (GFAP, 1:1000, chicken polyclonal, Abcam (ab4674)), NRG1 (1:300, rabbit polyclonal, Santa Cruz (sc-348)), and vesicular acetylcholine transporter (VAChT, 1:500, guinea pig polyclonal, Synaptic Systems (139 105)).

Once previously washed with PBS, sections were incubated for 1 h with a combination of appropriate secondary fluorescent antibodies labelled with one of the following fluorochromes (1/500): Alexa Fluor 488, DyLight 549, or DyLight 649 (Jackson Immuno Research Laboratories, West Grove, PA, USA). Finally, the spinal cord sections were labelled with blue fluorescent NeuroTrace Nissl staining (1:150; Molecular Probes) and mounted using an anti-fading medium containing 0.1 M Tris-HCl buffer (pH 8.5), 20% glycerol, 10% Mowiol, and 0.1% 1,4-diazabicyclo[2,2,2]octane. The slides were then examined under a FluoView FV-500 or FluoView FV-1000 Olympus Laser-Scanning Confocal Microscope (Olympus, Hamburg, Germany). The MNs were imaged after obtaining optical sections (0.5 or 1 μm) of cell bodies. For comparisons, slides from different animals and experimental conditions were processed in parallel for immunocytochemistry and subsequent imaging. The same scanning parameters were used for the acquisition of images corresponding to different experimental groups. Digital images were analysed using the FV10-ASW 3.1 Viewer (Olympus) and the ImageJ software (US National Institutes of Health, Bethesda, MD, USA). Immunolabelled profiles of the different protein markers were examined and then manually counted on the screen for each MN soma. The area and perimeter of MN somata and both microglial and astroglial profiles physically close to MNs were also manually measured. The digital images were edited using the FV10-ASW 3.1 Viewer (Olympus) and Adobe Photoshop CS4 (Adobe Systems Inc., San Jose, CA).

### Electron Microscopy and Ventral Root Axon Counting

Animals were perfused with 2% PFA and 2% glutaraldehyde in 0.1 M PB (pH 7.4) for conventional electron microscopy. Dissected tissues and the ventral roots were postfixed for 24 h at 4 °C in the above fixative solution, and, if needed, they were sectioned at 200 µm using a vibratome. The tissues were postfixed in 1% OsO4 for 2 h and then contrasted with 0.5% uranyl acetate for 30 min. Samples were processed for Embed 812 (Electron Microscopy Sciences, Hatfield, PA, USA) epoxy resin according to standard procedures. Semithin transversal sections (1 µm thick) were stained with Richardson stain and imaged using an Olympus ×60/1.4NA PlanApo oil immersion objective (Olympus) and a DMX 1200 Nikon (Tokyo, Japan) digital camera. Ultrathin sections were counterstained with Reynold’s lead citrate. All observations were performed on a transmission electron microscope JEOL JEM 1010 (Akishima, Tokyo, Japan).

### SOD1 Dot and Western Blotting

Frozen lumbar spinal cords were fragmented and homogenized using an electric homogenizer (Tissue Grinder) in ice-cold radioimmunoprecipitation assay (RIPA) lysis buffer (150 mM NaCl; 1% NP-40; 0.5% Na-deoxycholate; 0.1% SDS; 50 mM Tris-HCl [pH 7.4]), supplemented with protease inhibitor (Sigma-Aldrich, cat # P8340) and PhosSTOP (Roche). Homogenized samples were centrifuged at 800×*g* for 10 min at 4 °C. The protein concentrations of the supernatants were determined by BIO-RAD Micro DC protein assay (BIO-RAD, Laboratories, Inc.). For SOD1 dot blotting, 25 µg of protein were loaded in the wells of the Bio-dot protein blotting (BIO-RAD) and transferred by gravity filtering to a nitrocellulose membrane. Dot blot membranes were blocked in 3% BSA in Tris-buffered saline pH 8 (TBS), filtering it through the wells by gravity. For SOD1 western blotting, samples containing 25 µg of protein were heated at 100 °C for 5 min with an equivalent volume of sample buffer (containing 8% SDS and 2% mercaptoethanol) and loaded onto a denaturing 10% sodium dodecyl sulphate-polyacrylamide gel. The proteins were electrotransferred to a nitrocellulose membrane in Tris-glycine-methanol buffer. The membrane was blocked for 1 h at RT in a blocking solution mixture of 5% nonfat dry milk, 0.1% Tween 20, and TBS. Immunodetection was done by incubating the membranes overnight at 4 °C with pan-SOD1 (1:1000, sheep, Calbiochem (574597)), mouse monoclonal anti-actin (1:5000, Sigma-Aldrich, cat. # A5441), and rabbit polyclonal anti-GAPDH (1:10,000, Abcam, (ab181603)), the latter used for loading controls. Membranes were washed in TBS and incubated with the appropriate peroxidase-conjugated secondary antibodies (1:20,000, Cell Signaling, (cat. # 7076)) for 60 min at RT, washed in TBS tween (TBST), and visualized using the ECL Prime Western blotting Detection Reagent detection kit (GE Healthcare), as described by the manufacturer. Quantification of spot densities was performed using a Chemi-Doc MP Imaging System (BIO-RAD Laboratories, Inc.).

### NRG1 Signalling Western Blotting

Sucrose homogenization buffer (320 mM Sucrose, 10 mM Tris (pH 7.4), 1 mM NaHCO3, and 1 mM MgCl2) with protease and phosphatase inhibitors (Roche) was used for protein extraction. Proteins were separated on 8% SDS-polyacrylamide gels and blotted onto PVDF (Hybond-P, Invitrogen). Membranes were incubated with primary antibodies: actin mouse 1:1000 (Millipore), GAPDH mouse 1:1000 (Invitrogen), ErbB2 rabbit 1:500 (Cell Signaling), p-HER2/ErbB2 (Tyr877) rabbit 1:500 (Cell Signaling), ErbB4 rabbit 1:500 (Abcam), p44/42 MAPK (Erk1/2) rabbit 1:1000 (Cell Signaling), p-p44/42 MAPK (Erk1/2) (Thr202/Tyr204) rabbit 1:1000 (Cell Signaling), and Neuregulin-1a/b1/2 rabbit 1:250 (Santa Cruz), overnight at 4 °C. Membranes were washed 3× in 1× TBS-Tween and incubated in secondary antibodies (Alexa Fluor goat anti-mouse 680 1:10,000 (Invitrogen), Alexa Fluor goat anti-mouse 790 1:10,000 (Invitrogen), Alexa Fluor goat anti-rabbit 680 1:10,000 (Invitrogen), Alexa Fluor goat anti-rabbit 790 1:10,000 (Invitrogen)) for 1 h at RT, developed in an ODYSSEY DLx (LI-COR). Densitometric analysis of band intensities was carried out using ImageJ software.

### Phenotypical Score and Behaviour Analysis

Behavioural tests were performed every 10 days starting at post-natal (P) day 90 until P160. When they reached their end stage (ES), animals were euthanised. ES is determined by the Righting Reflex (RR) test. In the ES, mice that are placed on their sides cannot right themselves to the sternum in 30 s. The following behavioural tests were performed: (1) clinical score, (2) catwalk, and (3) rotarod. A clinical score (CS) test is a visual observation of the phenotypical condition of the animal. When the animal is not visibly affected, it receives a CS of 4. When paralysis of the first hindlimb appears, the CS is 3. The time the animals achieve this value is considered the disease onset. A CS of 2 is assigned when second-limb paralysis occurs. Finally, when the animal shows signs of symmetrical paralysis, the CS is 0 [34]. CatWalk XT (Noldus, Wageningen, Netherlands) was used to assess the motor profile of mice. This test consists of an enclosed walkway on a glass plate that is traversed by a mouse from one side of the walkway to the other. Data showing the gait pattern are acquired using an electronic device and analysed with the appropriate software. As the software showed some limitations for the study of later stages of a neurodegenerative disease like ALS, the interpodal distance was measured until the animal was able to complete the test. The rotarod (Ugo-Basile, Gemonio, Italy) test was performed to evaluate mouse motor performance. Animals were placed onto the rod, which turned at 4 rpm. The test was performed 3 times for each individual, and the longest latency without falling was recorded, with an arbitrary cut-off time of 180 s.

### Statistical Analysis

The data are expressed as means ± SEM. The statistical analysis was assessed by a Student’s *t*-test or by one- or two-way analysis of variance (ANOVA) followed by a post hoc Bonferroni’s test. The level of significance was established at *p* ≤ 0.05. GraphPad Prism 6 software was used for statistical analysis and graph presentations of data.

## Results

### Axonal Phenotype of Spinal MN from Double-Transgenic NRG1-SOD1^G93^^A^ Mice

**Fig. 1:**
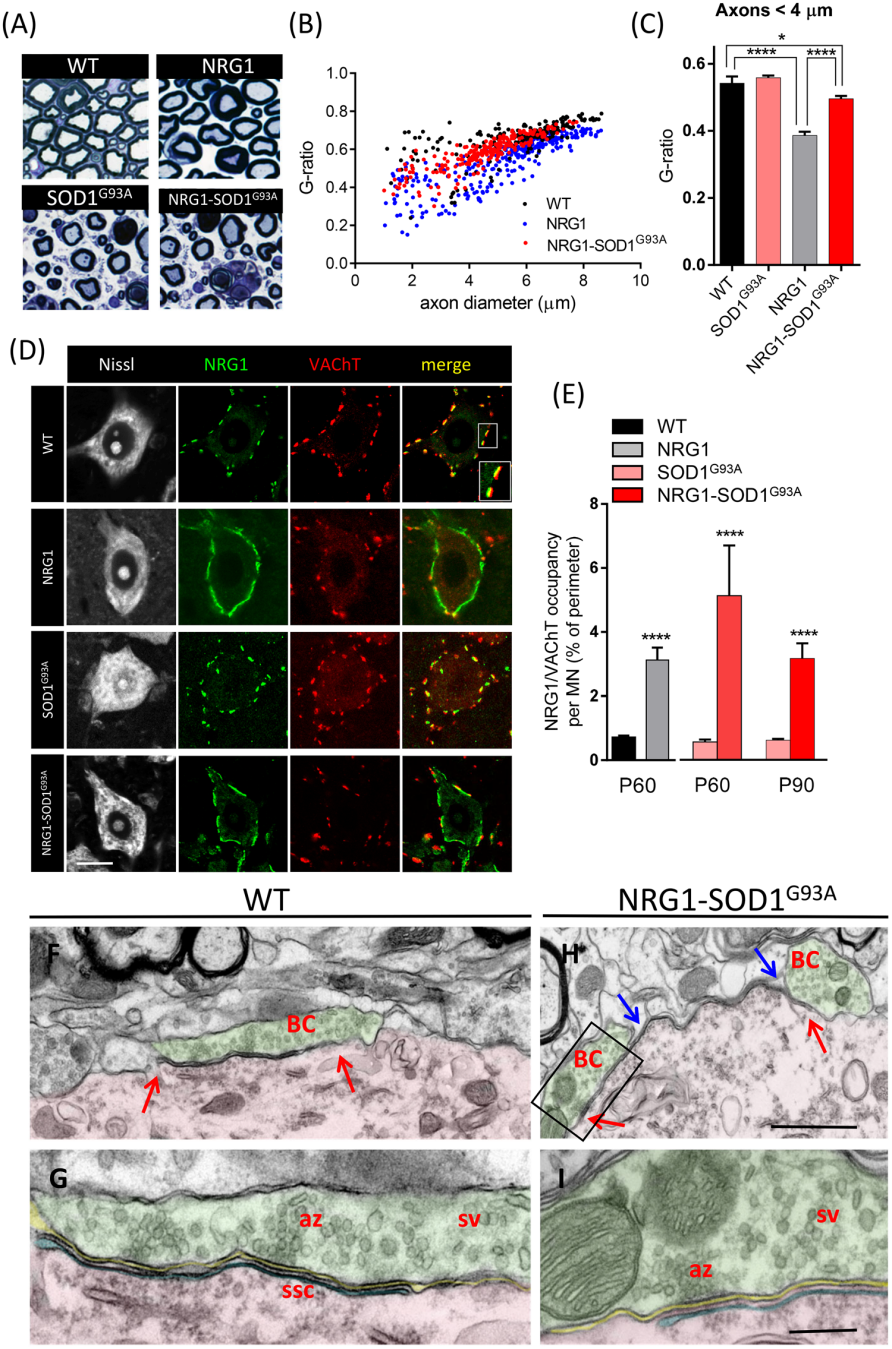
Phenotypical characterization of MNs in the double transgenic (SOD1^G93^^A^-NRG1) line. **a–c** Myelin thickness and *g* ratio measured on sections of L4 ventral roots at P60 (**a**); the hypermyelination of the large-sized axonal population was observed in NRG1 and NRG1-SOD1^G93A^ mutants with respect to WT (**b**, **c**). **d**, **e** MNs labelled for Nissl (grey), NRG1 (green), and VAChT (red), showing the association of afferent cholinergic terminals (C-boutons) with postsynaptic clusters of NRG1 in WT (see the dissociation between red and green signals in the inset). The NRG1 clusters at the periphery of MN somata are enlarged in both NRG1 and SOD1^G93A^-NRG1 mutants, whereas the VAChT-positive presynaptic counterpart of C-boutons remains unchanged. The extension of NRG1 clusters in MN somata was quantified in relation to VAChT at P60 and P90, as shown in **e**; note the tendency to reduce the NRG1 type III genetic-dependent enlargement of peripherally clustered NRG1 in P90 ALS MNs. Ultrastructural morphology of C-boutons (BC) in P60 WT (**f**, **h**) and SOD1^G93A^-NRG1 MNs (**g**, **i**). The presynaptic compartment (shaded in green) apposed to a MN surface (shaded in red) is filled with synaptic vesicles (sv) clustered at the active zones (az). A subsurface cistern (shaded in blue and delimited by arrows) is visible closely adjacent to the C-bouton postsynaptic membrane in WT (**f**, **g**) but extended beyond the presynaptic terminals in NRG1-SOD1^G93A^ (**h**, **i**). For nerve measurements, the sample size was as follows: WT, *n* = 39; NRG1, *n* = 76; NRG1-SOD1^G93A^, *n* = 70; *n* = axon number from 3 animals in every condition. For NRG1/VAChT clusters: *n* = 10–25 MNs from 3 animals in every condition. Scale bar: 10 µm (**c**), valid for the rest of the panel; 1 µm (**f**), valid for (**g**); 250 nm (**h**), valid for **i**. *p < 0.05; ****p < 0.0001

Hypermyelination of peripheral axons is a hallmark of NRG1 type III overexpressing mice [28, 29]. Thus, we first examined if the myelination-promoting function of NRG1 type III is preserved under conditions of SOD1^G93^^A^ overexpression in double transgenic NRG1-SOD1^G93A^ mice. Ventral root axons were still hypermyelinated in SOD1^G93A^-NRG1 double transgenic mice; however, hypermyelination was reduced, as revealed by *g* ratio analysis (Fig. 1A–C). Thus, SOD1^G93A^ overexpression interferes with NRG1-mediated myelination, perhaps as a consequence of presymptomatic disturbances in axonal transport described in SOD1^G93A^ mice [35].

**Fig. 2:**
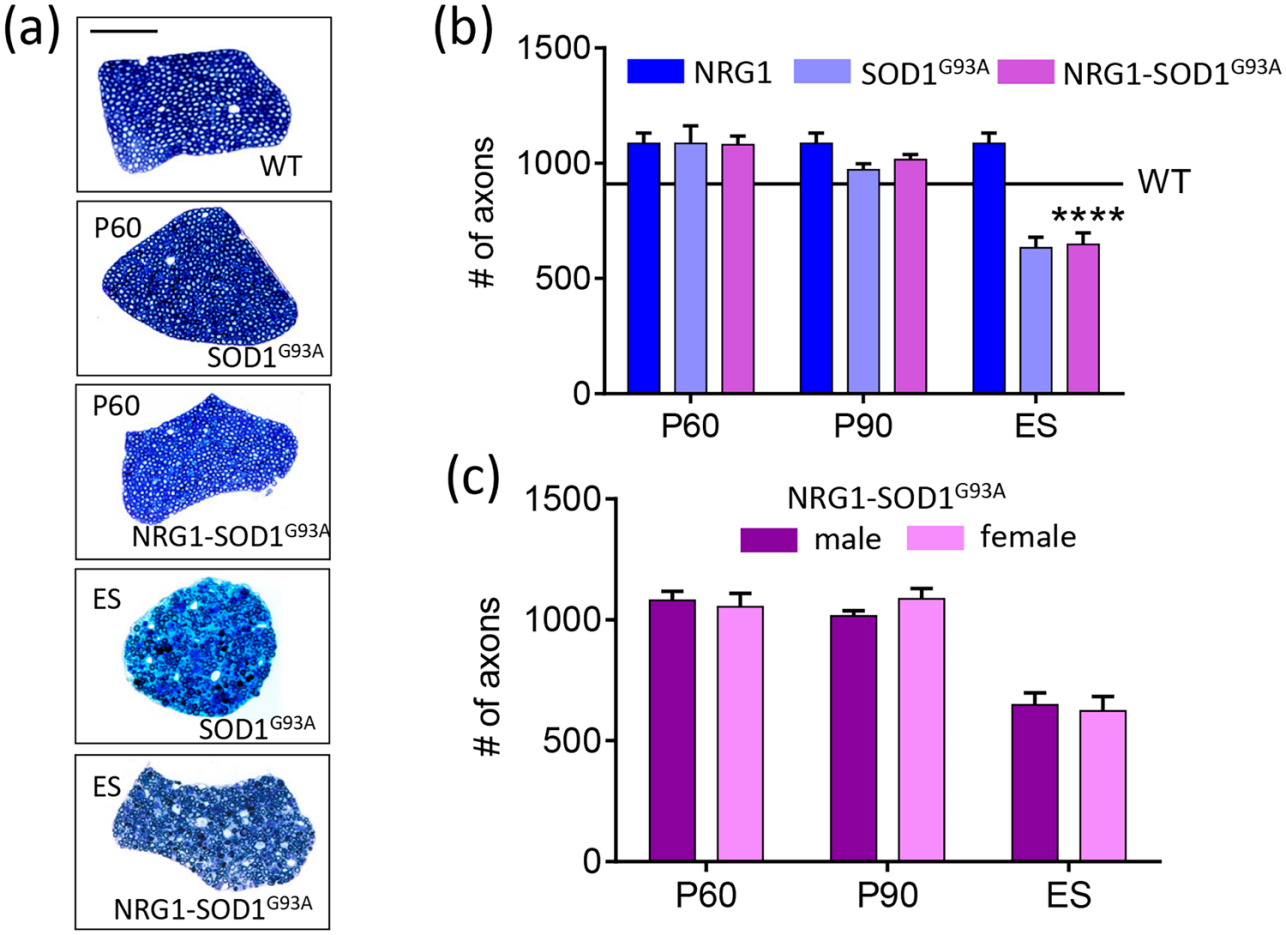
A number of apparently healthy axons quantified in transversal semithin sections of L4 ventral roots is shown in (**a**). Measures were done at different time points of the disease progression in the indicated genotypes (**b**) and segregated by sex in the double transgenic animals (**c**). ES, end stage. Sample size: WT, *n* = 900; NRG1, *n* = 1000; SOD1^G93^^A^, *n* = 600–1000; NRG1-SOD1^G93A^, *n* = 650–1000, *n* = axon numbers from 2 to 3 animals in every condition. Scale bar: 50 µm. ****p < 0.0001

It is well established that mutant SOD1^G93^^A^ expression does not impair myelination of peripheral nerves at early presymptomatic stages but that dramatic degenerative changes appear later in motor axons during disease progression [36, 37]. Thus, we next analyzed ventral nerve roots to detect if peripheral motor nerve degeneration observed in ALS was also seen under conditions of NRG1 type III overexpression. Axonal loss in ventral roots, which indicates MN death [36], and the pattern of nerve degeneration in L4 ventral roots at the end stage were similar in both SOD1^G93A^ and SOD1^G93A^-NRG1 mice, with no sex differences (Fig. 2). We conclude that promoting hypermyelination by NRG1 does not prevent MN death or the progression of the dramatic ALS-related motor axon pathology.

### MN Degeneration in Double-Transgenic NRG1-SOD1^G93^^A^ Mice

Both NRG1 type III [14, 38] and SOD^G93^^A^ are prominently expressed in the soma of spinal MN. To obtain a framework for the investigation of possible interactions between NRG1 type III and SOD^G93A^-mediated pathomechanisms in the MN soma, we first compared NRG1 type III expression in wild-type (WT) and transgenic mouse lines. Western blotting showed no difference in NRG1 expression in the spinal cord of SOD1^G93A^ mice and confirmed an increased NRG1 type III expression in NRG1 transgenic and NRG1-SOD1^G93A^ double-transgenic mice compared to WT; as expected, SOD1 signal on WB was highly increased in SOD1^G93A^ and NRG1-SOD1^G93A^ (Suppl. Fig. 1A, G). NRG1 immunoreactivity in MNs of adult WT was postsynaptically concentrated, adjacent to VAChT-positive puncta (Fig. 1D), which correspond to presynaptic cholinergic afferent terminals (C-boutons). At the postsynaptic site, NRG1 is co-clustered with other signalling molecules, such as S1R and Kv2.1 potassium channels (Suppl. Fig. 2), in ER-derived subsurface cisterns (SSC), in agreement with previous reports [13, 14, 17].

Overexpression of NRG1 type III in the MN of transgenic mice resulted in its accumulation in SSC. We previously reported that NRG1 accumulation is associated with an enlargement and reduplication of SSC-like membranes, as well as expanded expression of other proteins normally associated with SSC, such as S1R and Kv2.1 [30]. These SSC-related changes were also detected in double-transgenic NRG1-SOD1^G93^^A^ mice (Fig. 1D–I).

**Fig. 3:**
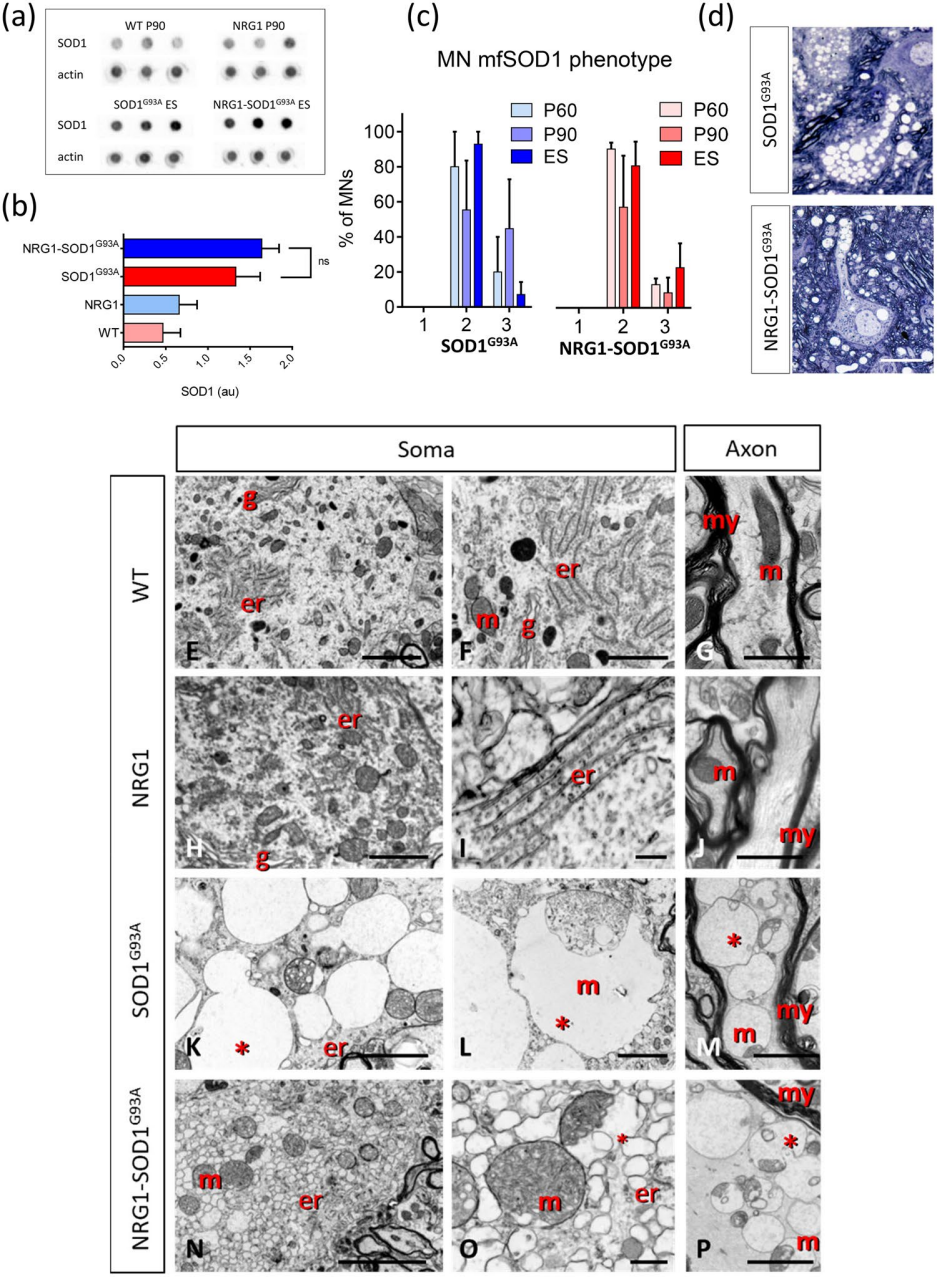
SOD1 aggregation and vacuolar degeneration in NRG1-SOD1^G93^^A^ mice. **a**,** b** Similar aggregation of mutant SOD1 protein in spinal cord extracts from SOD1^G93A^ and SOD1^G93A^-NRG1 mice as detected by the filter trap assay quantified in (**b)**, (*n* = 3). **c** Distribution of mfSOD1 phenotypes [42] in spinal cord ventral horn of SOD1^G93A^ and NRG1-SOD1^G93A^ mice immunostained with the conformational-specific anti-SOD1 antibody C4F6; note the tendency, although not significant, for reduced appearance of type 3 phenotype (accumulation of somatic mfSOD1) at disease onset (P60-90) in NRG1-SOD1^G93A^ mice, whereas the proportion of type 2 phenotype (accumulation of mfSOD1 in the neuropil) is similar in both genotypes. **d** Vacuolar degeneration in SOD1^G93A^ and NRG1-SOD1^G93A^ MNs; note the extensive vacuolar formation in the soma and neuropil in SOD1^G93A^ and its reduction in the soma of NRG1-SOD1^G93A^ MNs, in which vacuoles are concentrated in MN processes and neuropil. **E–P** Ultrastructural morphology of MN somata and axons of P60–P90 MNs from WT, SOD1^G93A^, and NRG1-SOD1^G93A^ mice (er, endoplasmic reticulum; g, Golgi apparatus; m, mitochondria; my, myelin; *mitochondria-derived vacuoles). Note the presence of massive mitochondrial-derived giant vacuoles in the soma of a SOD1^G93A^ MN; compared with the soma of NRG1-SOD1^G93A^ MN, in which formation of mitochondrial-derived vacuoles appears to be limited; instead, there is an extensive microvacuolar degeneration of ER origin. Note the comparable accumulation of mitochondrial-derived vacuoles in axons from SOD1^G93A^ and NRG1-SOD1^G93A^ mice. Sample size for mfSOD1 phenotypes: SOD1^G93A^, *n* = 7–92; and NRG1-SOD1^G93A^, *n* = 12–87; *n* = MN number from 3 animals at P60, P90, and ES. Scale bars: 20 µm (**d**); 2.5 µm (valid for **H**, **K**, **L**) (**E**); µm (valid for **G**, **J**, **M**, **P**) (**F**); 5 µm (**n**)

Altered proteostasis leading to an accumulation of misfolded (mf) SOD1 aggregates is a hallmark of familial ALS involving SOD1 and also seems to play a role in sporadic ALS [39–41]. By measuring the amount of aggregated SOD1 trapped on a filter membrane using a dot blotting procedure, we found no significant differences between spinal cord extracts from SOD1^G93^^A^ and NRG1-SOD1^G93A^ mice (Fig. 3A, B).

Cellular expression of mfSOD1 in SOD1^G93^^A^ mice can also be analysed by means of conformation-specific anti-SOD1 antibodies, which allow for the monitoring of cell-specific pathological processes [18]. Based on mfSOD1 detection in spinal cord sections of SOD1^G93A^ mice, we recently described three phenotypes of MN pathology that can be observed, starting at the early presymptomatic stages of ALS [42]. These phenotypes correlate well with the vacuolar degeneration that can be observed either restricted to MN processes (phenotype 2) or extended into MN somata (phenotype 3), whereas phenotype 1 represents the absence of mfSOD1 immunoreactivity in ventral horns. When the relative amount of these phenotypes was analysed in double transgenic mice (Fig. 3C), we found that mfSOD1 expression in axons and dendrites was not affected by NRG1 type III overexpression. However, the pathomorphological alteration leading to the extensive vacuolization in MN soma outlining phenotype 3 was reduced in double transgenic mice, such that at P90, 44.7 ± 12.3% (*n* = 3 mice) of MN soma from SOD1^G93A^ mice displayed a robust mfSOD1 signal and vacuolated pattern, whereas these alterations were only observed in 8.3 ± 4.8% (*n* = 3 mice) of MNs in double transgenic mice.

**Fig. 4:**
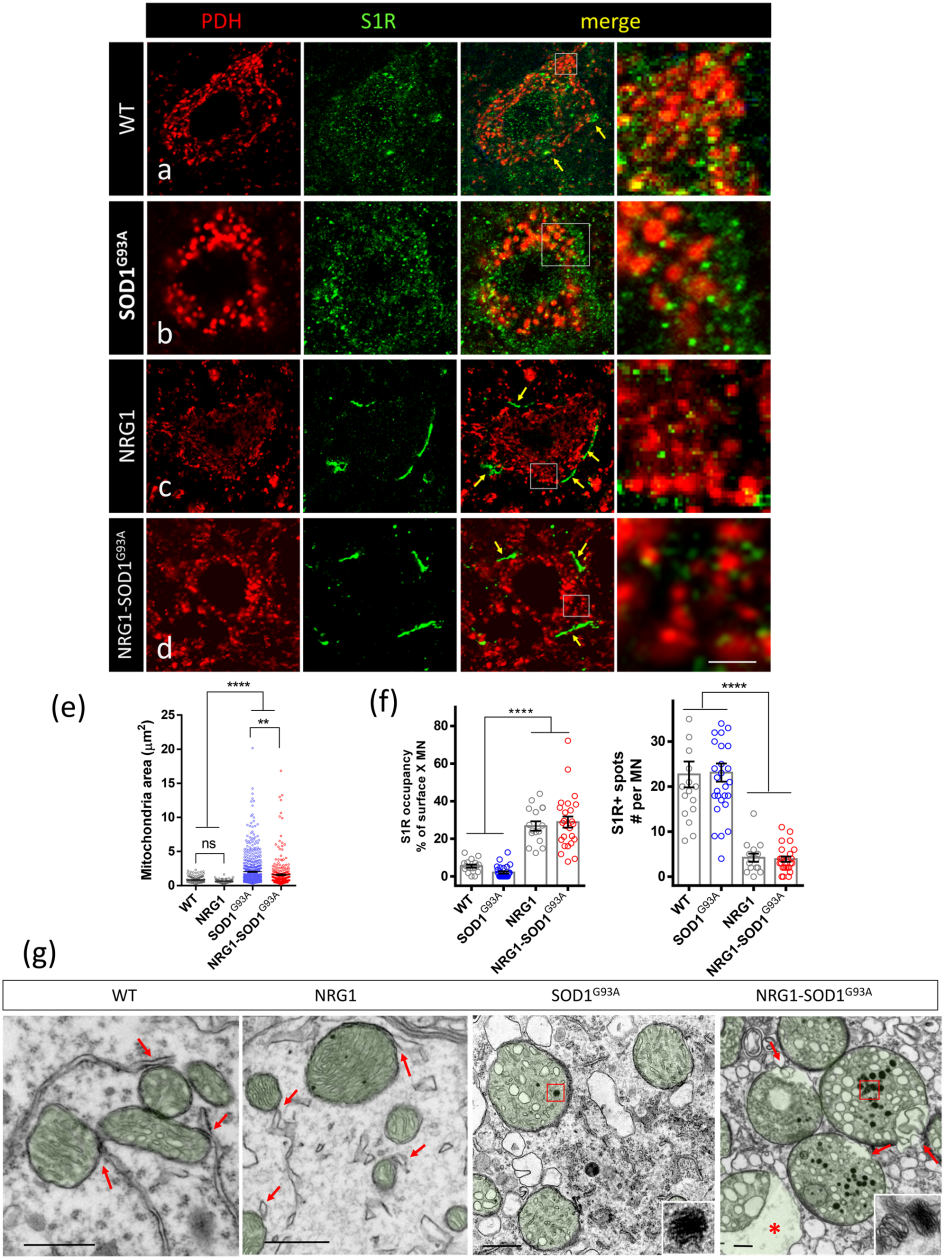
Changes in S1R distribution and mitochondrial pathology in SOD1^G93^^A^ and NRG1-SOD1^G93A^ mutant mice. **a–d** Mitochondria are visualized by PDH (red) in combination with S1R (green) in P60 MNs. In WT, S1R is found clustered near the MN surface (arrows) and also distributed in small puncta in the cytoplasm of WT and SOD1^G93A^ MNs. Most puncta are in close relationship with mitochondria, probably corresponding to MAMs (shown in (**a**) and (**b**)). In the NRG1 overexpressors, the S1R clusters close to the MN surface (arrows) are expanded, and this was also observed in the double transgenic SOD1^G93A^-NRG1 mice (**c**, **d**). **e** Quantification of mitochondrial size in MNs of P60 mice with the indicated genotype; note that the mitochondrial enlargement seen under SOD1^G93A^ expression is reduced under a NRG1 type III overexpression background. **f** Quantification of the internal S1R spots and external occupancy of S1R clusters in P60 MN somata at the indicated genotypes. **g** Ultrastructural morphology of mitochondria in P60 MNs. The sites of interaction of mitochondria with ER membranes (MAMs) are clearly seen in WT and NRG1 MNs (arrows). Note that the vesicular transformation of the cristae and membranous inclusions (inset) are similarly observed in altered mitochondria in SOD1^G93A^ and SOD1^G93A^-NRG1 MNs. Sites presumably corresponding to the disrupted MAMs are indicated by arrows. The asterisk indicates an image of constrained vacuolar formation in a mitochondrion of NRG1-SOD1^G93A^ MN. Sample size: WT, *n* = 193; NRG1, *n* = 71; SOD1^G93A^, *n* = 645; NRG1-SOD1^G93A^, *n* = 294; *n* = mitochondria number from 3 animals at each condition; WT, *n* = 18; NRG1, *n* = 15; NRG1-SOD1^G93A^, *n* = 25; *n* = MN number from 3 animals at each condition. **p < 0.005; ****p < 0.0001; ^##^*p* < 0.005 (in (**d**), asterisk with respect to NRG1 and number sign with respect to SOD1^G93A^). Scale bars: 10 µm (**d**); inset = 2 µm (valid for **a–d**); 0.5 µm (**g**)

Most vacuoles found in MNs of the ALS SOD1^G93^^A^ mouse model result from a massive expansion of the mitochondrial outer membrane [42]. Accordingly, changes in the structure and organization of mitochondria were analysed in more detail. By electron microscopy, a similar number of vacuoles were present in the axons and dendrites of SOD1^G93A^ and NRG1-SOD1^G93A^ mice, consistent with the unaltered abundance of the type 2 mfSOD1 phenotype in both lines. In contrast, mitochondrial vacuoles were less abundant in the MN soma of NRG1-SOD1^G93A^ mice (Fig. 3 D). Thus, despite showing a plethora of structural alterations, the expansive growth of the outer membrane leading to enlarged vacuoles was reduced in the MN somata of double transgenic animals (Fig. 3E–P). The delimitation of mitochondrial profiles by PDH immunostaining confirmed that NRG1 type III overexpression in NRG1-SOD1^G93A^ mice was associated with a significant constraint of the vast mitochondrial enlargement, a feature typically observed in SOD1^G93A^ MNs (Fig. 4A–E). Some MN somata from double transgenic animals (but not from NRG1 mice) displayed extensive microvacuolar degeneration rather than the giant vacuoles of mitochondrial origin seen in SOD1^G93A^ mice (Fig. 3E–P). These smaller vacuoles seem to derive from massive vesicular fragmentation of the ER, and ER-derived vesicles in SOD1^G93A^ mice could be a source of an accretive growth of the outer mitochondrial membrane leading to the formation of giant vacuoles [42]. Thus, we hypothesized that ER-derived vesicles in NRG1-SOD1^G93A^ mice may remain longer in a stationary state due to a blockade of their fusion with mitochondrial external membranes and that sites where both organelles physiologically interact, referred to as mitochondria-associated ER membranes (MAMs), could play a role in this process.

One protein relevant in this context is the Sigma1 receptor (S1R) [43, 44]. Subcellular S1R expression was unaltered in presymptomatic SOD1^G93^^A^ mice when compared to wildtype, but S1R accumulated close to the MN surface in NRG1-SOD1^G93A^ mice, in concomitance with NRG1 type III overexpression and increased SSC biogenesis (Fig. 4A–D). The subsurface S1R accumulation was associated with a decreased number of cytoplasmic S1R-positive puncta, which, in part, may represent MAMs (Fig. 4F). This change is most likely directly caused by NRG1 type III overexpression since it was also observed in NRG1 transgenics, independently of SOD1^G93A^ expression. Moreover, a biochemical analysis found no evidence for increased activation of ErbB2 receptors or MAP kinase, a major downstream signalling pathway, in the spinal cord of NRG1 and NRG1-SOD1^G93A^ mice (Suppl. Fig. 1E, F). Thus, it is conceivable that increased SR1 targeting to the MN subsurface is mediated by NRG1 type III accumulation in SSC independent of ErbB receptor activation and results in a reduction of SR1 protein availability at other cytoplasmic sites. As a consequence, SR1 depletion at MAMs of NRG1-SOD1^G93A^ mice could interfere with mitochondria–ER interactions required for the formation of large vacuoles observed in the MN somata of SOD1^G93A^ mice. Consistent with this notion, we observed a focal accumulation of vesicles at the mitochondrial surface that presumably corresponds to disorganized MAMs (Fig. 4G).

Profound mitochondrial alterations other than extreme vacuolar degeneration were observed in SOD1^G93^^A^ and NRG1-SOD1^G93A^ MNs. These comprised prominent changes in inner membrane organization with an electron-dense matrix, completely unstructured cristae, and rare outlines including vesicle-like structures. Some electron-opaque membrane inclusions were also observed in these mitochondria in both models (Fig. 4G). These inclusions, in association with the other described ultrastructural changes, are indicative of severe calcium disturbances. In conjunction, our data suggest a specific effect of NRG1 in reducing vacuolar enlargement by modulating MAMs but not in other mitochondrial functions.

### Astrogliosis and Microgliosis During ALS Progression in SOD1^G93^^A^ Mice Are Not Attenuated Under NRG1 Type III Overexpression

**Fig. 5:**
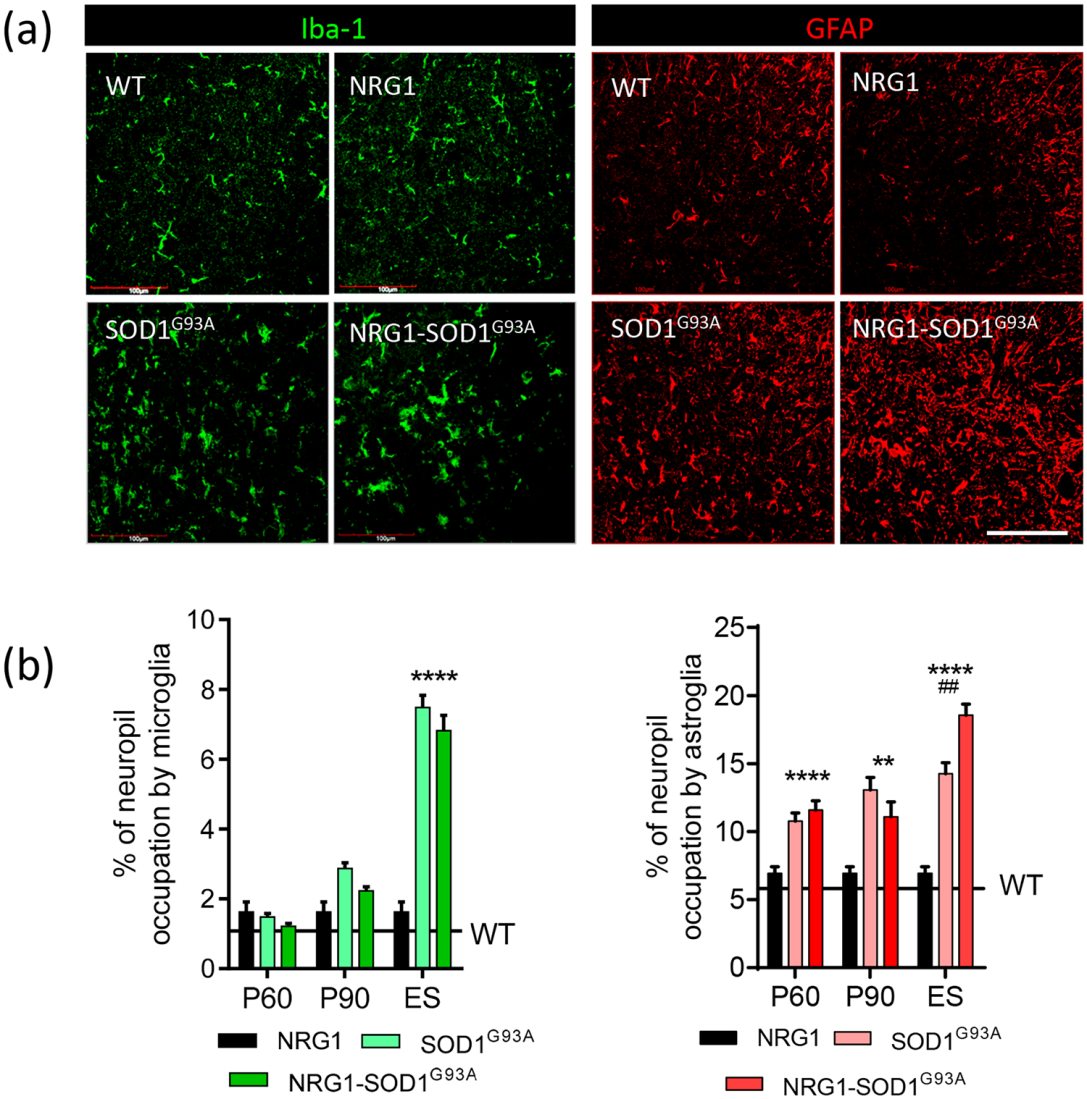
SOD1^G93^^A^ ALS-related neuroinflammatory glial reactions are not altered in SOD1^G93A^-NRG1 mice. **a** Representative image of mouse ($\sim$ P120) ventral horn spinal cord sections after Iba1 (green) and GFAP (red) immunostaining for microglia and astroglia, respectively, of the indicated genotypes. **b** Quantification of microglia and astroglia in ventral horn at different ages and genotypes as indicated. Sample size: NRG1, *n* = 42; SOD1^G93A^, *n* = 35–46; NRG1-SOD1^G93A^, *n* =27–42; *n* = ventral horn neuropil from 3 animals at each condition. **p < 0.005; ****p < 0.0001; ^##^*p* < 0.005 (asterisk with respect to NRG1 and number sign with respect to SOD1^G93A^). Scale bar: 100 µm (valid for all panels)

Conflicting data have emerged from recent studies regarding the impact of altered NRG1 signalling on glial responses during ALS progression in SOD1^G93^^A^ mice [23, 26, 27]. When measured from presymptomatic (P60) to ES of disease progression, astroglial and microglial activation were not broadly different in SOD1^G93A^ and NRG1-SOD1^G93A^ mice, as shown by GFAP and Iba1/CD68 immunostaining, respectively; only at the ES of the disease, astrogliosis was slightly higher in NRG1-SOD1^G93A.^ (Fig. 5; Suppl. Fig. 3). Note that the sole overexpression of NRG1 type III already produced a small increase in basal glial occupancy in the spinal cord ventral horn, consistent with a role of NRG1 signalling in microglial activation [45] and our own recent findings of increased microglia in NRG1 mutants [30].

### NRG1 Type III Overexpression Does Not Affect Survival of SOD1^G93^^A^ Mice but Accelerates Disease Onset and Worsens Motor Phenotype

**Fig. 6:**
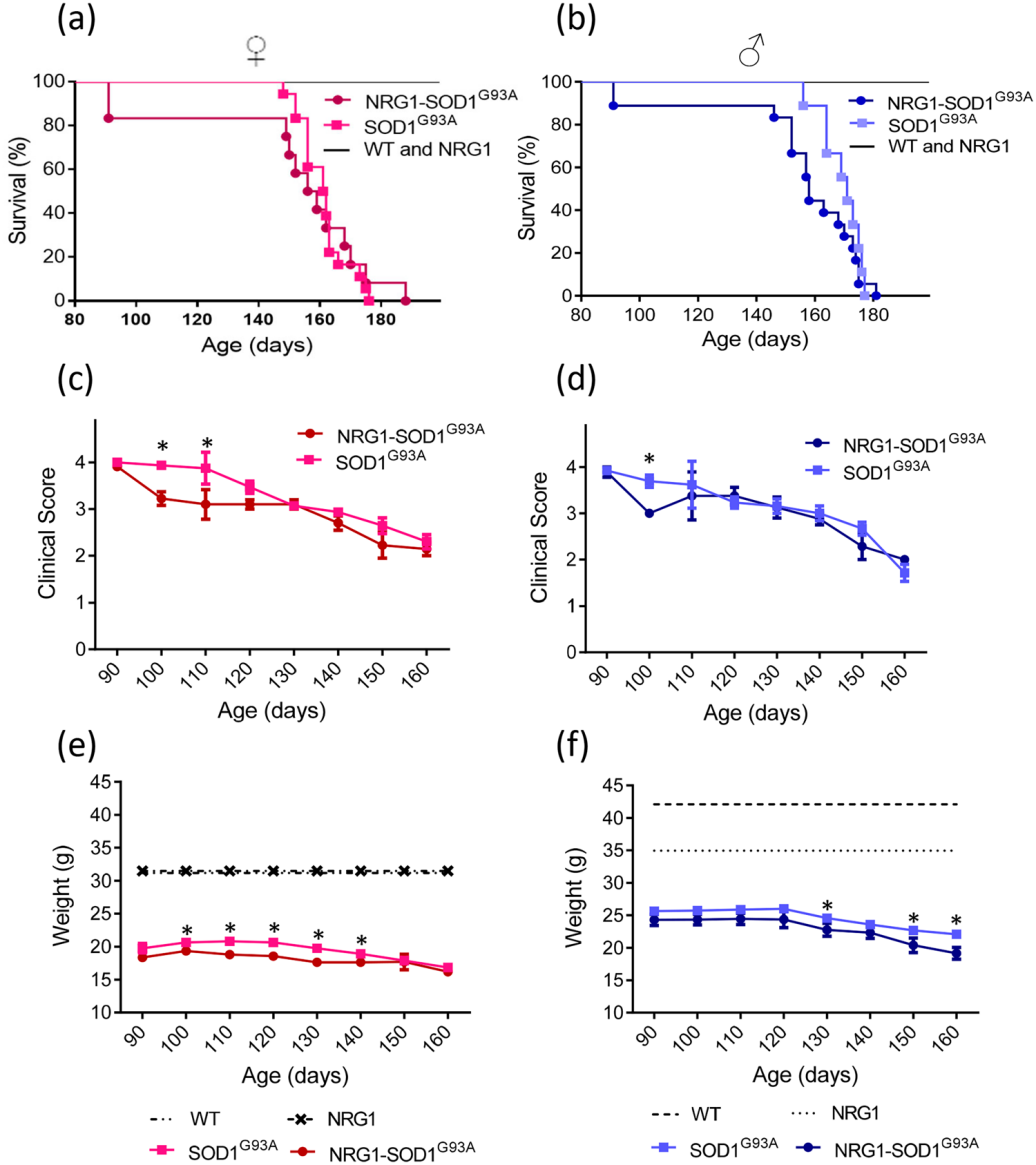
NRG1 type III overexpression does not extend survival in SOD1^G93^^A^ mice. Kaplan–Meier analysis of the proportion of survival of female NRG1-SOD1^G93A^ mice compared to female littermates SOD1^G93A^ (**a**) and male NRG1-SOD1^G93A^ mice compared to male littermates SOD1^G93A^ (**b**). The mean survival ages for NRG1-SOD1^G93A^ and SOD1^G93A^, respectively, were as follows: 150.9 days ± 8.71 (*n* = 12) and 161.2 days ± 1.84 (*n* = 18) for females (**a**) and 155.2 days ± 5.98 (*N* = 18) and 169.4 days ± 2.32 (*N* = 9) for males (**b**). NRG1 type III overexpression accelerates disease onset in SOD1^G93A^ females (**c**) and males (**d**). Disease onset starts when CS reaches a value of 3. *p < 0.05; **p < 0.01; ***p < 0.001 (Student’s *t*-test comparing the two genotypes in each time point). The disease course of the mice was evaluated based on body weight alterations, reflecting significant denervation-induced muscle atrophy in double transgenic mice compared to their littermate SOD1^G93A^ controls (**e**, females; **f**, males); *p < 0.05; **p < 0.01; ***p < 0.001 (Student’s *t*-test comparing the two genotypes in every time point). Number of analysed mice: *N* = 12 for NRG1-SOD1^G93A^ and *N* = 18 for SOD1^G93A^ (female animals); *N* = 18 for NRG1-SOD1^G93A^ and *N* = 9 for SOD1^G93A^ (male animals) (**c–f**). *p* < 0.001 (Student’s *t*-test comparing the two genotypes at each time point)

To examine the impact of NRG1 type III overexpression on the SOD1^G93^^A^ mouse clinical phenotype, we tested survival and motor performance. To identify potential sex differences, male and female mice were independently examined. Analysis revealed no significant differences in lifespan and motor function in NRG1 transgenic mice compared to WT (Fig. 6). No significant differences in lifespan were also observed in female (Fig. 6A) or male (Fig. 6B) NRG1-SOD1^G93A^ mice when compared to sex-matched SOD1^G93A^ littermates. However, using a clinical score test [34], we observed an earlier onset in NRG1-SOD1^G93A^ mice compared to SOD1^G93A^ mice, both for females (Fig. 6C) and males (Fig. 6D).

Since denervation-induced muscle atrophy highly correlates with a decrease in body weight, weight loss has been considered a reliable indicator of disease course [46]. Therefore, we evaluated the disease course based on body weight changes. Even considering that NRG1 transgenic males had lower weight compared to WT males, we found significant differences during disease progression for both females and males (Fig. 6E).

**Fig. 7:**
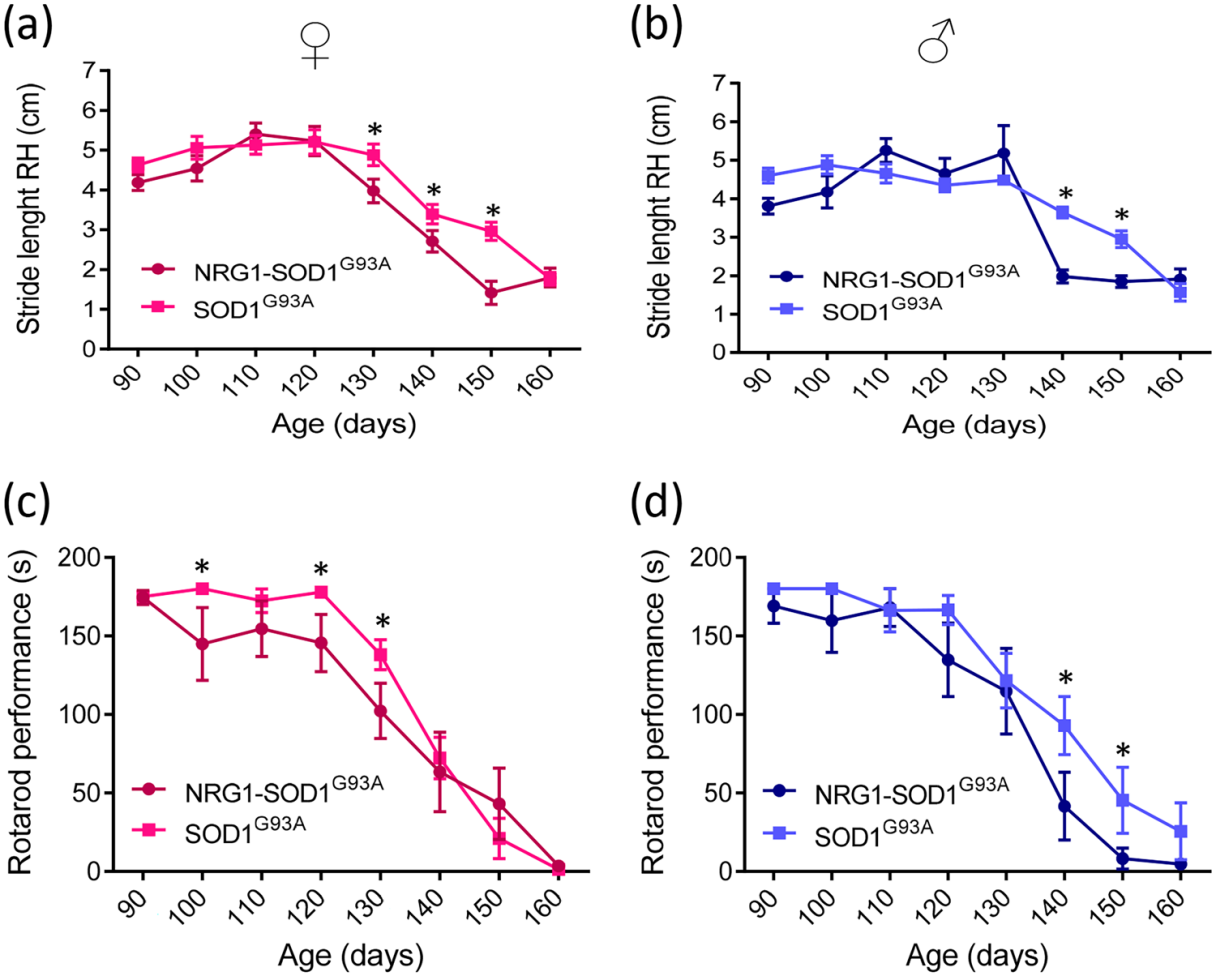
Catwalk analysis shows a reduced right hindlimb stride length of females (**a**) and males (**b**) associated with the NRG1 type III overexpression in SOD1^G93^^A^ mice. *p < 0.05; **p < 0.01; ***p < 0.001 (Student’s *t*-test comparing the two genotypes at every time point). Average rotarod test latency to fall times of females (**c**) and males (**d**) NRG1-SOD1^G93A^ and SOD1^G93A^ mice. *p < 0.05; **p < 0.01; ***p < 0.001 (Student’s *t*-test comparing the two genotypes at each time point). For the motor test, the number of analysed female animals were *N* = 12 for NRG1-SOD1^G93A^ and *N* = 18 for SOD1^G93A^, and, for males, *N* = 18 for NRG1-SOD1^G93A^ and *N* = 9 for SOD1^G93A^ (Student’s *t*-test, comparing the two genotypes in each time point)

Motor performance was examined using the Catwalk XT analysis system. The stride length of the right and left hind limbs was measured at different time points. Reduced stride length for the right hindlimb in NRG1-SOD1^G93^^A^ females (Fig. 7A) and males (Fig. 7B) was observed at different time points when compared to sex-matched SOD1^G93A^ littermates. A similar stride length reduction in the left hindlimb was found in double transgenic mice compared to SOD1^G93A^ mice (Suppl. Fig. 4). These results were consistent with data from motor function analysis using the rotarod test. These results showed that the average latency to fall was reduced in NRG1-SOD1^G93A^ double transgenics compared to SOD1^G93A^ mice. Thus, we conclude that the overexpression of NRG1 worsens the motor performance in the SOD1^G93A^ background.

## Discussion

Several studies have evaluated the potential value of manipulating NRG1 signalling as a new target for ALS therapy in SOD1^G93^^A^ mice. For this, two strategies were used: virus-mediated delivery of NRG1 type III into the spinal cord to promote a supplementary action of this trophic factor [23, 47] or, conversely, blockade of NRG1 receptor activation by intrathecal administration of a specific antagonist [27]. Surprisingly, both approaches, although apparently divergent, resulted in an improvement of some clinical and pathological aspects of the disease. Here, we took advantage of a genetically well-defined overexpression of NRG1 type III in a transgenic mouse line to evaluate its effects on ALS outcome in a double transgenic NRG1-SOD1^G93A^ mouse model. We found that most clinical and pathological ALS-related disease hallmarks were largely unaltered in NRG1-SOD1^G93A^ double-transgenic mice.

The accumulation of mfSOD1, MN degeneration, including mitochondrial pathology, and the glial-mediated neuroinflammatory response are the main pathological hallmarks of ALS progression in SOD1^G93^^A^ mice. There was no significant improvement in most of these pathologies in the NRG1-SOD1^G93A^ model. However, the extent of vacuolar degeneration in MNs with mfSOD1 accumulation was decreased upon NRG1 type III overexpression, mainly because of a reduced mitochondrial expansion during the formation of vacuoles in MN somata. This could result from altered ER-mitochondrial interactions caused by the change in intracellular membrane organization, which occurs when NRG1 type III is overexpressed [30]. Intracellular membrane remodelling in NRG1-SOD1^G93A^ mice involved drastic changes in the expression and distribution of a set of C-bouton proteins to synaptic and extrasynaptic sites. This included S1R, a component of the protein complex present at ER-mitochondrial membrane contact sites. These contacts assemble using tethering proteins and have key functions in the organelles involved, mainly related to lipid trafficking and Ca [2]^+^ transfer. The distribution of S1R is dramatically altered when NRG1 type III is overexpressed in neurons [30]. Moreover, S1R appears to be involved in ALS pathology, as S1R mutations are causative of some forms of familial ALS [48] and are also involved in SOD1-linked ALS [49]. The pharmacological activation of S1R prolongs the survival of SOD1 ALS mice [50–52], and S1R inactivation or loss determines MN degeneration [53], suggesting SR1 as a promising target for further preclinical ALS research.

Although we found a reduced formation of giant mitochondria-derived vacuoles in MN somata of NRG1-SOD1^G93^^A^ mice, this was not observed in MN axons or dendrites, which were affected by vacuolar degeneration similar to SOD1^G93A^ mice. These MN soma-targeted effects could result from NRG1 accumulation restricted to somatic SSC. Thus, based on our biochemical and histological findings, we speculate that NRG1 overexpression-associated changes in membrane dynamics in MN cell bodies are independent of ErbB2/ErbB4 signalling but are rather linked to SSC accumulation of full-length NRG1 type III, including its C terminus, which could serve as an interaction site with other proteins, such as S1R.

Although neuromuscular junctions (NMJs) were not analysed in detail, it should be noted that NRG1 type III overexpression itself has an important impact on the activity of terminal Schwann cells, thereby regulating the formation and stability of NMJs during development and, probably, throughout life [54]. As NMJ dismantlement is an early event inherent to the ALS phenotype [55], upregulation of NRG1 signalling in NRG1-SOD1^G93^^A^ mice could provide additional stress to ALS-diseased NMJs that may contribute to earlier disease onset and deficits in motor function.

Taken together, in contrast to previous studies where NRG1 was shown to promote functional improvement in the SOD1^G93^^A^ background [26, 47], we found no evidence for amelioration in lifespan, disease onset, or motor function in NRG1-SOD1^G93A^ mice when compared with control SOD1^G93A^ mice. Conversely, we found that double-transgenic mice exhibit an earlier disease onset, faster disease progression, and a trend for a reduced lifetime. We argue that these data are convincing because they were obtained by using several behavioural tests in a large, sexually segregated cohort following the guidelines for preclinical research in ALS/MND [56]. These results are in concordance with the absence of any overt improvement in the histopathological changes linked to the disease. Nevertheless, we must leave open the possibility that, compared to persistent overexpression, modulating NRG1 levels in a more restricted temporal-spatial manner could be beneficial for the treatment of distinct aspects of MN degeneration in ALS.

### Supplementary Information

Below is the link to the electronic supplementary material.

Supplementary Fig. 1 (**A**) Western blotting of SC protein lysates indicates increased expression of NRG1 in adult NRG1-SOD1G93A and NRG1 transgenic mice, compared to SOD1G93A and wildtype mice (age P151-P174). Using an antibody directed against the C-terminal NRG1 domain. GAPDH was used as loading control. (**B**) Densitometric quantification of full-length NRG1. Integrated density values were normalized to GAPDH. n=4 each; **p < 0.01, ***p < 0.001, ****p < 0.0001; One-way ANOVA. (**C**) Western Blot analysis of SC protein lysates (age P151-P174) for ErbB2 and pErbB2 expression. (**D**) Densitometric quantification of pErbB2 bands. Integrated density values were normalized to ErbB2 and β-actin loading control; n=3 each; ns, p > 0.05; One-way ANOVA (**E**) Western blot analysis of SC protein lysates (age P151-P174) for MAPK and pMAPK expression. (**F**) Densitometric quantification of pMAPK bands. Integrated density values were normalized to MAPK and β-actin loading control; n=4 each; ns, p > 0.05; One-way ANOVA. (**G**) Western blotting of spinal cord extracts reacted with a pan-SOD1 antibody demonstrating the overexpressed protein in both SOD1G93A mutants (PDF 1962 KB)

Supplementary Fig. 2 Kv2.1 potassium channels are clustered on MN surface at C-bouton synaptic sites in close association with NRG1 clusters. A spinal cord MN is shown after simultaneous fluorescent labelling of NRG1 (green), Kv2.1 (red) and VAChT (white) to delimitate C-type synapses. Nissl-staining of the multilabelled MN (blue) is also depicted. Scale bar = 10 µm (PDF 2998 KB)

Supplementary Fig. 3 Iba1 immunostaining (red) was combined with CD68 (green) for lysosome detection in activated microglia at the indicated genotypes. (**A**) Representative images of spinal cord ventral horn. At the end of each row an enlarged detail of double labeled microglia profiles is shown. Scale bar = 50 mm (20 mm for detailed panels). (**B**) The proportion of the area delimited by Iba1 that was occupied by CD68 was measured in each condition. Data in the graph are presented as mean ± SEM from 5-8 sections from each condition (representing 1649-5344 Iba1-positive profiles); ns= not significant, *p < 0.5, ***p < 0.001, Studenthatt-test. Bar= 50 µm (in enlarged panels = 20 µm) (PDF 6967 KB)

Supplementary Fig. 4 Motor performance was examined using the Catwalk XT analysis system. Stride length of left hindlimb was measured at different time points. Reduced stride length for the left hindlimb in SOD1G93A-NRG1 females (**A**) and males (**B**) was observed at different time points when compared to sex matched SOD1G93A littermates (PDF 401 KB)

## Data Availability

The datasets used and/or analysed are available from the corresponding author upon reasonable request.

## Abbreviations

ALS: amyotrophic lateral sclerosis
ANOVA: analysis of variance
az: active zones
CNS: central nervous system
CNTF: ciliary neurotrophic factor
CS: clinical score
EGF: epidermal growth factor
ES: end stage
GDNF: glial-derived neurotrophic factor
GFAP: glial fibrillary acidic protein
Iba1: ionized calcium-binding adapter molecule 1
IGF-1: insulin-like growth factor 1
LIT: literature
MAMs: mitochondria-associated ER membranes
mfSOD1: misfolded SOD1
MNs: motor neurons
NRG1: neuregulin-1
P: postnatal
PB: phosphate buffer
PBS: phosphate-buffered saline
PFA: paraformaldehyde
RIPA: radioimmunoprecipitation assay
RR: righting reflex
RT: room temperature
SOD1: superoxide dismutase 1
SSC: subsurface cistern
TBS: tris-buffered saline
TBST: tris-buffered saline tween
VAChT: vesicular acetylcholine transporter
VEGF: vascular endothelial growth factor
WT: wild type
