# Supplementary figures and images for: Persistent NRG1 Type III Overexpression in Spinal Motor Neurons Has No Therapeutic Effect on ALS-Related Pathology in SOD1G93A Mice
Source: Neurotherapeutics. 2023 Sep 21;20(6):1820–34. doi: 10.1007/s13311-023-01424-x (PMC10684470; doi:10.1007/s13311-023-01424-x)

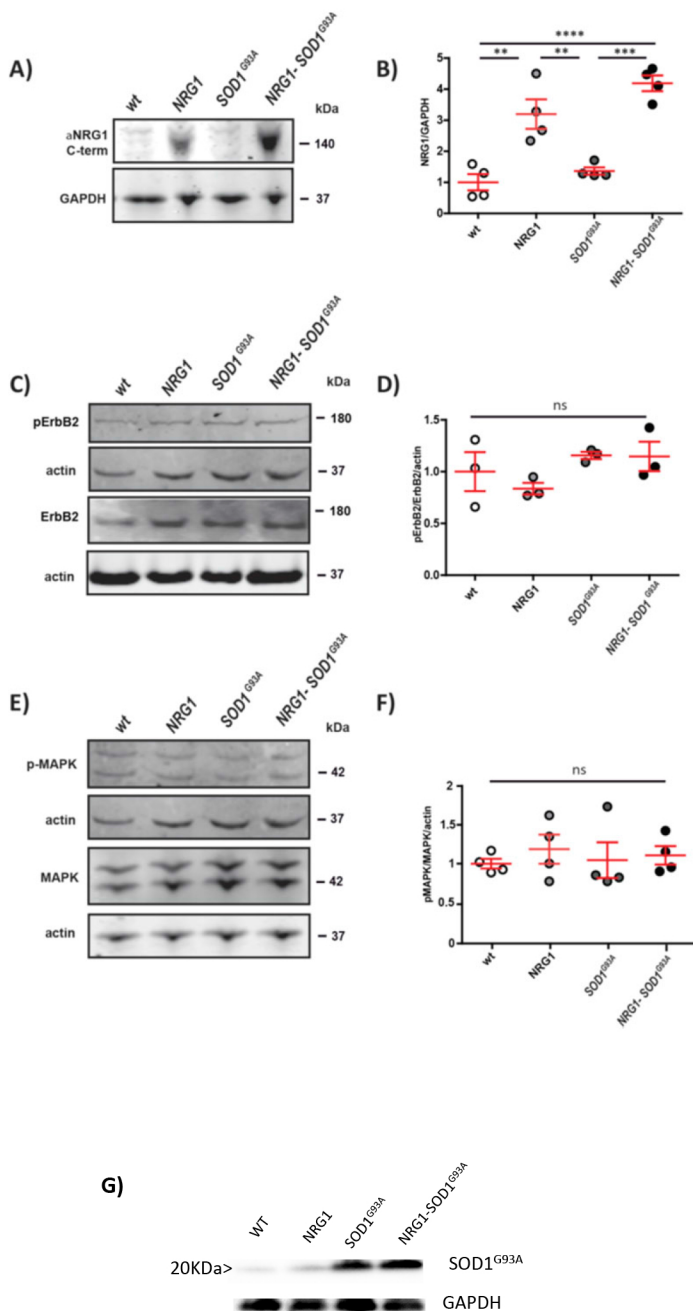

Supplement: Supplementary file 1 — Supplementary Fig. 1 (A) Western blotting of SC protein lysates indicates increased expression of NRG1 in adult NRG1-SOD1G93A and NRG1 transgenic mice, compared to SOD1G93A and wildtype mice (age P151-P174). Using an antibody directed against the C-terminal NRG1 domain. GAPDH was used as loading control. (B) Densitometric quantification of full-length NRG1. Integrated density values were normalized to GAPDH. n=4 each; **p < 0.01, ***p < 0.001, ****p < 0.0001; One-way ANOVA. (C) Western Blot analysis of SC protein lysates (age P151-P174) for ErbB2 and pErbB2 expression. (D) Densitometric quantification of pErbB2 bands. Integrated density values were normalized to ErbB2 and β-actin loading control; n=3 each; ns, p > 0.05; One-way ANOVA (E) Western blot analysis of SC protein lysates (age P151-P174) for MAPK and pMAPK expression. (F) Densitometric quantification of pMAPK bands. Integrated density values were normalized to MAPK and β-actin loading control; n=4 each; ns, p > 0.05; One-way ANOVA. (G) Western blotting of spinal cord extracts reacted with a pan-SOD1 antibody demonstrating the overexpressed protein in both SOD1G93A mutants (PDF 1962 KB) [file 13311_2023_1424_MOESM1_ESM.pdf]

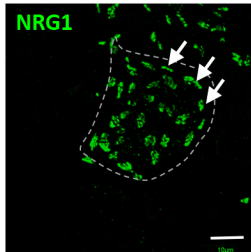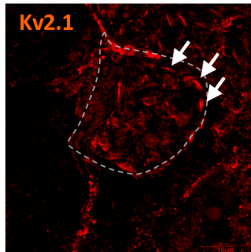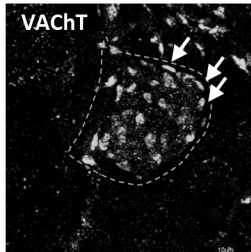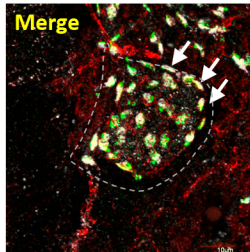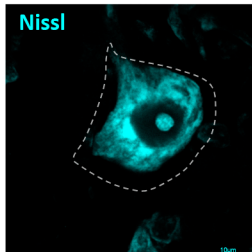

Supplement: Supplementary file 2 — Supplementary Fig. 2 Kv2.1 potassium channels are clustered on MN surface at C-bouton synaptic sites in close association with NRG1 clusters. A spinal cord MN is shown after simultaneous fluorescent labelling of NRG1 (green), Kv2.1 (red) and VAChT (white) to delimitate C-type synapses. Nissl-staining of the multilabelled MN (blue) is also depicted. Scale bar = 10 µm (PDF 2998 KB) [file 13311_2023_1424_MOESM2_ESM.pdf]

A

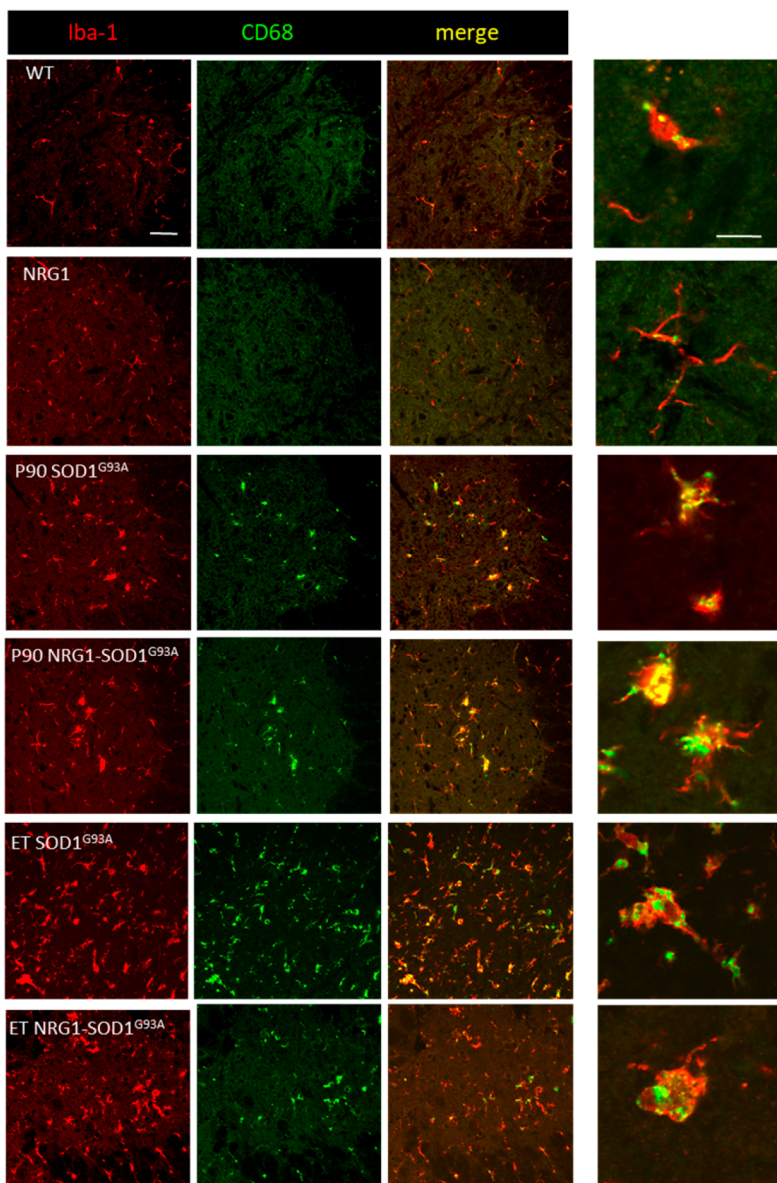

B

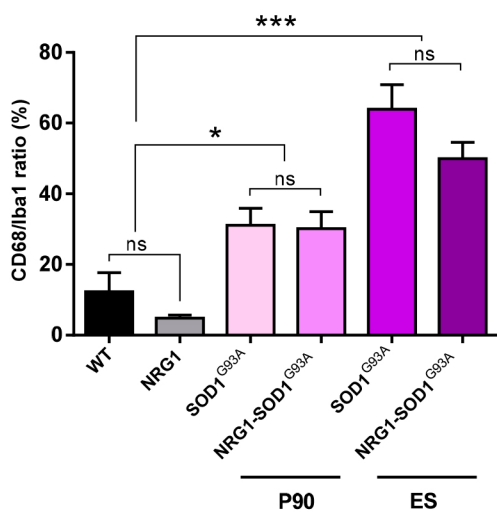

Supplement: Supplementary file 3 — Supplementary Fig. 3 Iba1 immunostaining (red) was combined with CD68 (green) for lysosome detection in activated microglia at the indicated genotypes. (A) Representative images of spinal cord ventral horn. At the end of each row an enlarged detail of double labeled microglia profiles is shown. Scale bar = 50 mm (20 mm for detailed panels). (B) The proportion of the area delimited by Iba1 that was occupied by CD68 was measured in each condition. Data in the graph are presented as mean ± SEM from 5-8 sections from each condition (representing 1649-5344 Iba1-positive profiles); ns= not significant, *p < 0.5, ***p < 0.001, Studenthatt-test. Bar= 50 µm (in enlarged panels = 20 µm) (PDF 6967 KB) [file 13311_2023_1424_MOESM3_ESM.pdf]

**A**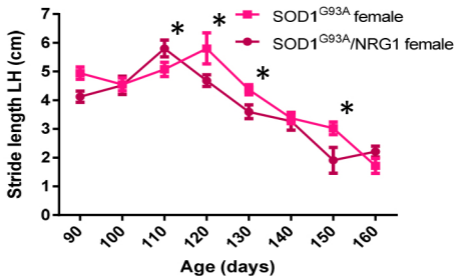**B**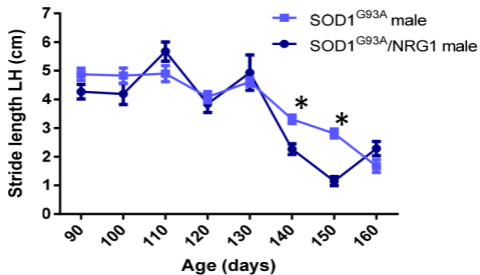

Supplement: Supplementary file 4 — Supplementary Fig. 4 Motor performance was examined using the Catwalk XT analysis system. Stride length of left hindlimb was measured at different time points. Reduced stride length for the left hindlimb in SOD1G93A-NRG1 females (A) and males (B) was observed at different time points when compared to sex matched SOD1G93A littermates (PDF 401 KB) [file 13311_2023_1424_MOESM4_ESM.pdf]
